# OD-YOLO: Robust Small Object Detection Model in Remote Sensing Image with a Novel Multi-Scale Feature Fusion

**DOI:** 10.3390/s24113596

**Published:** 2024-06-03

**Authors:** Yangcheng Bu, Hairong Ye, Zhixin Tie, Yanbing Chen, Dingming Zhang

**Affiliations:** 1 School of Computer Science and Technology, Zhejiang Sci-Tech University, Hangzhou 310018, China; 2021329621211@mails.zstu.edu.cn (Y.B.); tiezx@zstu.edu.cn (Z.T.); cyb@zstu.edu.cn (Y.C.); 2021329621200@mails.zstu.edu.cn (D.Z.); 2 KeYi College, Zhejiang Sci-Tech University, Shaoxing 312369, China

**Keywords:** YOLO, remote sensing, small object detection

## Abstract

As remote sensing technology has advanced, the use of satellites and similar technologies has become increasingly prevalent in daily life. Now, it plays a crucial role in hydrology, agriculture, and geography. Nevertheless, because of the distinct qualities of remote sensing, including expansive scenes and small, densely packed targets, there are many challenges in detecting remote sensing objects. Those challenges lead to insufficient accuracy in remote sensing object detection. Consequently, developing a new model is essential to enhance the identification capabilities for objects in remote sensing imagery. To solve these constraints, we have designed the OD-YOLO approach that uses multi-scale feature fusion to improve the performance of the YOLOv8n model in small target detection. Firstly, traditional convolutions have poor recognition capabilities for certain geometric shapes. Therefore, in this paper, we introduce the Detection Refinement Module (DRmodule) into the backbone architecture. This module utilizes Deformable Convolutional Networks and the Hybrid Attention Transformer to strengthen the model’s capability for feature extraction from geometric shapes and blurred objects effectively. Meanwhile, based on the Feature Pyramid Network of YOLO, at the head of the model framework, this paper enhances the detection capability by introducing a Dynamic Head to strengthen the fusion of different scales features in the feature pyramid. Additionally, to address the issue of detecting small objects in remote sensing images, this paper specifically designs the OIoU loss function to finely describe the difference between the detection box and the true box, further enhancing model performance. Experiments on the VisDrone dataset show that OD-YOLO surpasses the compared models by at least 5.2% in mAP50 and 4.4% in mAP75, and experiments on the Foggy Cityscapes dataset demonstrated that OD-YOLO improved mAP by 6.5%, demonstrating outstanding results in tasks related to remote sensing images and adverse weather object detection. This work not only advances the research in remote sensing image analysis, but also provides effective technical support for the practical deployment of future remote sensing applications.

## 1. Introduction

Due to the ongoing advancements in remote sensing technologies, the utilization of Remote Sensing Images (RSIs) has expanded significantly, including in the detection of lake surface water [1], applications in grain production [2] and landslide research [3]. In recent years, the capabilities of neural network models have continuously improved. And, computer vision technology has advanced significantly, resulting in object detection methods that are vastly different from those of the past. The models of deep learning can extract deeper features from remote sensing images, showing great potential for applications in the recognition of remote sensing image targets [4].

However, the characteristics of remote sensing images also lead to several problems as follows: Firstly, remote sensing images include satellite images and images taken by unmanned aerial vehicles (UAVs), which differ significantly from ground-level images. What is more, remote sensing images exhibit rich diversity, capturing subjects across a variety of scales, perspectives, and forms. This diversity makes object detection more challenging. Secondly, remote sensing images have very large differences in object size, and these images cover both small and large targets. This difference in scale increases the complexity of the object detection task. Further, remote sensing images have a complicated background condition, which introduces a new consideration of environmental factors for object detection. The textures and colors of the objects in the images can closely resemble those of the background and complicate their differentiation. Therefore, it raises the level of challenge in detecting. Simultaneously, images captured through remote sensing often suffer from diminished object details and insufficient information, posing challenges to precise object detection. Finally, the weather has a great influence on remote sensing images; for example, there is a big difference between rainy days and sunny days in terms of the ability to capture objects, and bad weather may lead to blurred images or noise. The combination of these factors compounds the challenges associated with detecting targets in RSI.

This paper introduces the OD-YOLO model framework in response to the challenging conditions of remote sensing images, aiming to enhance its object detection capabilities within remote sensing imagery tasks. We have designed a DRmodule, which enhances feature extraction for geometric structures. According to the Feature Pyramid Network (FPN) of YOLOv8 [5], this paper introduces the Dynamic Head module, which effectively improves the feature fusion capability across layers in the FPN. This paper proposed a new OIoU loss function. The OIoU can more accurately describe the localization of the targets in images. These improve the performance of the model, which shows an improvement of about 5.2% in mAP50 and 4.4% in mAP75 compared to recentadvanced methods.

The main contributions of this paper are as follows:

1. In this paper, we propose the OD-YOLO framework for target detection in remote sensing scene captured by unmanned aerial vehicles.
2. An enhanced feature extraction Detection Refinement module (DRmodule) and OIoU loss function are employed to improve the model’s capacity to gather features from small objects and detect them.
3. Experiments with a remote sensing object detection dataset prove that the OD-YOLO effectively boosts the performance in detect objects in remote sensing scenes.

The rest of the paper is organized as follows: Section 2 offers an overview of related work in the field. Section 3 provides a thorough explanation of the method we propose. The results from comparative and ablation studies are detailed in Section 4. Section 5 explores the disadvantages of our approach and offers ideas for improvements in the future. Finally, Section 6 provides a summary of the findings and conclusions drawn from this research.

## 2. Related Work

### 2.1. YOLO Model

As deep learning grows, CNN has made great achievements, and YOLO [6] is one of the most popular models in CNNs. The YOLO model boasts an extensive array of applications in industry due to its simple structure, lightweight network and excellent detection results. Zhukov et al. [7] used the YOLO model with an attention mechanism and achieved better results in rail lines defect detection. Zhao et al. [8] have improved the YOLOv5 by making it lightweight to enable the rapid detection of sewer defects. Chen et al. [9] used the YOLO model to achieve good results in suspicious object detection in millimeter wave images. Ding et al. [10] improved the YOLOv5 model’s capacity for global feature fusion by integrating the EPMS module. Tang et al. [11] improved the object detection ability of the YOLO model in complex traffic roads by introducing an RMA module. Wu et al. [12] proposed the SOD-YOLO model to achieve outstanding capacity in detecting mini-goal in the high-voltage transmission lines. Yuan et al. [13] effectively improved the precision in detecting defects of PCB by using the LW-YOLO model. Song et al. [14] proposed MED-YOLO to improve the model’s recognition ability in complex traffic scenes. Du et al. [15] developed the DSW-YOLO model for strawberry fruit recognition and successfully implemented it in a strawberry-picking robot, achieving relatively good results. Wan et al. [16] proposed the LGP-YOLO, which has led to considerable advancements in identifying surface defects on light guide plates.

In other methods, the ability of YOLO model’s feature extraction is mainly improved through the attention mechanism, which essentially assists the model to accurately pinpoint and identify the target objects. But mostly, these models ignore the neck structure feature structure. In this paper, we raise the capacity of the feature fusion of the FPN in the framework. To achieve this, our method uses the Dynamic Head [17] to improve the overall recognition precision.

### 2.2. Remote Sensing Object Detection

Over the past few years, as objects detection technology has continually evolved, significant progress has been made in object detection in remote sensing imagery. However, many problems persist in detecting small targets, which are difficult to identify. Du et al. [18] effectively tackled the issue of losing crucial feature information of small-scale targets during multiple downsampling by combining feature integration across multiple scales and integrating an attention module. Liang et al. [19] proposed an object identification approach based on a novel cloud collaboration and restructured convolutional architecture, which improves the model’s capacity in detecting remote sensing scenes in real time. Wu et al. [20] introduced the GNAM module, which combines several attention mechanisms to create a global normalized attention weight. This helps better utilize valuable information in the input feature channels and spatial dimensions, improving the effectiveness of the model in identifying remote sensing targets. Liu et al. [21] integrated a data augmentation algorithm with a highly efficient subspace attention module, while also fine-tuning the quantity of detection heads and refining the loss function. In a training period, Mai et al. [22] employed a dynamic dual-domain alignment (DDA) approach, which addresses possible mismatches in spatial and feature domains throughout the learning phase. Zhang et al. [23] introduced Drone-YOLO, which improves the capability of the model in understanding different scales features by utilizing the APFN [24] structure and RepVGG [25].

This paper proposes the DRmodule to tackle the difficulties of identifying small and diverse-shaped objects in remote sensing imagery. This module effectively lowers the likelihood of missing small objects and improves the model’s capabilities in identifying targets across remote sensing images.

### 2.3. Small Object Detection

In traditional machine learning tasks, detecting small objects has always been a challenging task because the features of small object images are not distinct, making them difficult for models to recognize. Attention mechanisms and improved loss functions are often used to promote the model’s effectiveness in the tasks of detecting small target. Mo et al. [26] incorporated multi-attention into YOLOv5 to yield improved outcomes in detecting small objects on airport runways. Yang et al. [27] merged an enhanced channel attention mechanism with a better version of E-ELAN [28] to introduce an upgraded YOLOv7 model, which is designed to identify small spots on grape leaves. Aibibu et al. [29] combined the strengths of various networks to improve the detection performance of small target vehicles. Liu et al. [30] utilized dynamic snake convolution [31] and introduced WISE-IoU [32] to boost the model’s effectiveness in detecting small traffic-related objects. Wang et al. [33] proposed a joint attribute soft-sharing and contextual local method to improve the model’s efficacy in re-identifying pedestrians.

In this paper, the OIoU loss function is used, which is a more precise and stable metric tailored to small target detection. This approach enhances the precision and resilience in detecting objects.

## 3. Proposed Method

This paper uses the YOLOv8n model as a foundational architecture and presents the OD-YOLO framework. We propose the DRmodule in the OD-YOLO framework, specifically designed to identify small objects in remote sensing images, aiming to improve the model’s feature extraction performance. Additionally, we incorporate the OIoU loss to boost the model’s ability to semantically express detection boxes of small objects. Our model also integrates a Dynamic Head to strengthen the feature fusion ability in the feature pyramid. OD-YOLO enhances feature representation, leading to improved recognition accuracy in identifying objects within remote sensing imagery tasks.

**Figure 1:**
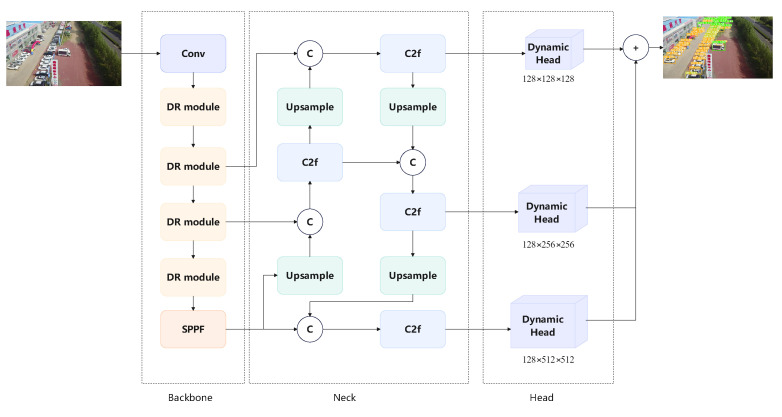
The framework of OD-YOLO. Four DRmodules are placed in the backbone for feature extraction. In the neck, features across scales are fused. Finally, detection targets are output using three dynamic heads of different scales.

As shown in Figure 1, three components are in the framework of the OD-YOLO: Backbone, Neck and Head. Before being fed into the network, the input image’s resolution will be adjusted to 640×640. Mosaic data augmentation stitches together four images using random cropping, brightness adjustments, and flipping. Feature Pyramid Network (FPN) [34] and Path Aggregation Network (PANet) [35] are used in OD-YOLO’s neck, and finally, the OD-YOLO uses Dynamic Head to boost feature fusion capability. At last, the model outputs detection boxes, confidence scores, and categories in the form of a Decoupled Head.

### 3.1. Detection Refinement Module

**Figure 2:**
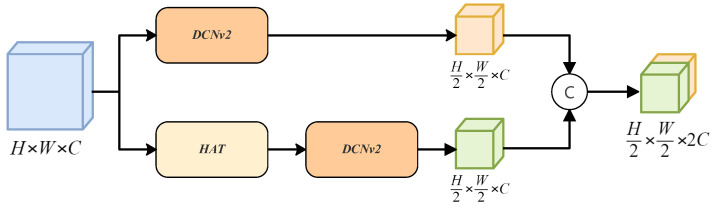
The structure of DRmodule. It integrates Deformable Convolutional Networks and Hybrid Attention Transformer for enhanced geometric feature extraction in remote sensing imagery.

Objects in remote sensing images may be relatively small. It is challenging for YOLO to detect samll targets because of the lack of specific information. To address these challenges, we designed a module called the Detection Refinement module (DRmodule), which is placed in the Backbone of the YOLO module to further enhance detection performance. The DRmodule enhances multi-scale expressive abilities and improves performance in low-resolution settings for detecting small targets in remote sensing images by integrating DCNv2 [36,37] and the Hybrid Attention Transformer [38]. As shown in Figure 2, the DRmodule uses DCNv2 to derive geometric features from the input features. Then, a Hybrid Attention Transformer (HAT) is applied for feature extraction before using DCNv2 again to extract deeper features. These two sets of features are concatenated together. By fusing features across scales and adapting to deformations, the DRmodule enhances the effectiveness of the model in handling complicated situations in the images of remote sensing, thus improving the precision and reliability of target detection.

#### 3.1.1. Deformable Convolutional Networks

In traditional CNN, convolution kernels have a very fixed structure, which results in poor feature extraction capability for certain geometric structures. DCNv2 [36,37] is an enhanced convolution technique capable of adaptively modifying the convolution kernel shape to more effectively accommodate the target’s deformation or multi-scale features. The scale of targets in remote sensing imagery can differ substantially, and DCNv2 can better capture features of targets at different scales. Objects in remote sensing images may undergo unstructured deformations, such as tilting or morphing. DCNv2 can handle these situations better by adapting the shape of its convolution kernels, thus boosting the precision of object detection.

For a DCNv2, in a 3×3 convolution, we assume that the relative positions of the convolutionally extracted features are as shown Equation (1).

(1) q={(−1,−1),(−1,0),...,(1,0),(1,1)}.

In DCNv2, each position of the convolution feature extraction has a position offset Δp and a weight coefficient Δm, learned from the preceding feature map. Therefore, the final feature output map is as shown Equation (2).

(2) $$y\left(p_{0}\right)=\sum_{p_{n}\in q}w\left(l_{n}\right)\cdot x\left(p_{0}+p_{n}+\Delta p_{n}\right)\cdot \Delta m_{n}$$

Here, $p_{0}$ denotes the value at each location on the output feature map *y*, $\Delta m_{n}$ is a decimal between [0, 1] used to represent the weight of that position. $p_{n}$ represents the point in the input feature map. $\Delta p_{n}$ denotes the offset learned from the earlier feature map.

DCNv2 dynamically adapts to each feature extraction position by introducing a deviation that enables dynamic feature extraction. It also incorporates a weight, allowing for varied feature expression based on each position’s unique characteristics. It can capture detailed information about the boundaries and complex shapes of target objects more accurately, especially useful for detecting objects that change shape. This approach effectively describes the geometric shapes of targets in remote sensing image recognition and boosts OD-YOLO’s capability to extract features from small objects.

#### 3.1.2. Hybrid Attention Transformer

Remote sensing object detection tasks face challenges such as highly complex backgrounds and multi-scale objects, which often lead to limitations in the recognition accuracy and generalization capabilities of traditional models. By using HAT’s advanced features, including its robust global and local information processing abilities and acute sensitivity to complex backgrounds, the model significantly enhances its capability to detect objects of different scales.

**Figure 3:**
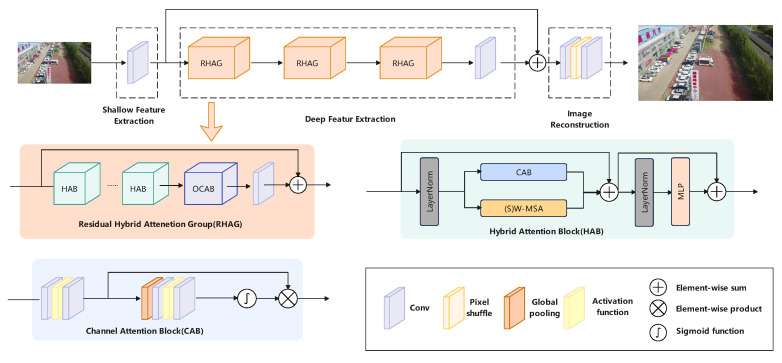
Diagram of the Hybrid Attention Transformer (HAT) in the DRmodule, showing how it extracts basic features, processes them with attention groups for detailed analysis, and then rebuilds the image to better capture object details for detection.

As shown in Figure 3, to derive shallow features from the image, HAT initially applies a convolution process, and it employs several RHAGs and a 3×3 convolution process for deriving deep feature. After that, the convolution layer and a Pixel Shuffle layer [39] is used for rebuilding the resolution, and then another 3×3 convolution layer is used to produce the ultimate image. The RHAG has several HAB layers; an OCAB layer and a 3×3 convolutional layer with a residual connection are used to achieve better reconstruction effects. This module comprises two principal elements: Window-based Self-Attention [40] and Channel Attention [41]. First, the input features are normalized, then processed using the window-based self-attention mechanism. This mechanism segments the features maps into local windows, and every window will calculate self-attention to capture the association information of local areas. Next, through the channel attention, more global features are introduced to calculate channel attention weights. This attention module utilizes global insights to weight the features, thus activating more pixels.

**Figure 4:**
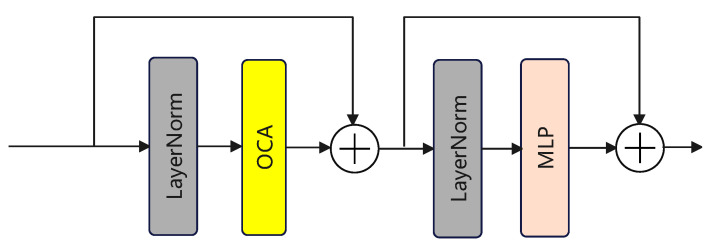
The Overlapping Channel Attention Block (OCAB) in the HAT, showing how it uses attention across different areas and channels to better detecting targets.

In HAT, the OCAB module is additionally proposed, as shown in Figure 4. Compared to the CAB module, it introduces an overlapping cross-attention layer to establish cross-connections between windows in window self-attention, enhancing the network’s representation ability.

**Figure 5:**
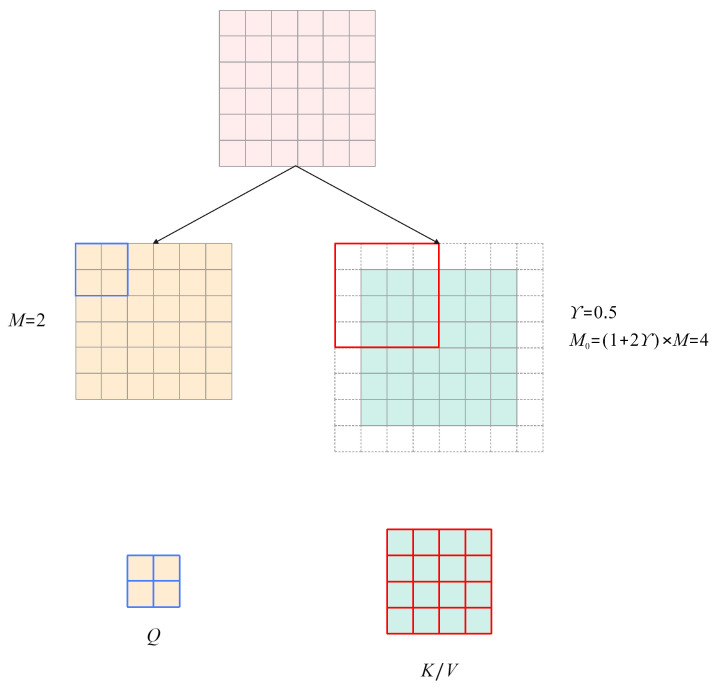
The Overlapping Cross-attention Layer.

As shown in Figure 5, the specific computation procedure resembles that of the W-SMA module [40], but in calculating the attention mechanism, zero-padding is applied to the original image during the computation of K/V, allowing for learning from the content in another window through a larger window. When a feature map is input into this layer, it is divided into several M∗M windows, which serve as the Query in the attention mechanism. At the same time, the original feature map undergoes zero-padding controlled by the parameter γ. The padded feature map is divided into $M_{0}*M_{0}$ feature maps, which serve as the Key/Value. The attention algorithm is as shown in Equation (3). The design of this module enables better utilization of the pixel information within a window for queries, thereby improving the model’s performance.

(3) $$Attention\left(Q,K,V\right)=SoftMax\left(QK^{T}/\sqrt{d}+B\right)V$$

Here, *d* stands for the dimension of *Q* and $K^{T}$, and *B* stands for the encoding of the position. The computation of Q,K,V is as described above. The calculation of attention is refered to in the literature [42].

Through incorporating the HAT module into the architecture of the OD-YOLO model, we boost the model’s capacity to process fine features in remote sensing images and markedly promote the model’s adaptability to the unique spatial variability of remote sensing images through HAT’s channel and window self-attention mechanisms. This improvement boosts the precision in the detection of the remote sense and strengthens the model’s reliability against complex backgrounds and varied target sizes.

### 3.2. Dynamic Head

A major challenge in detecting objects in remote sensing is handling target detection across multiple scales and against complex backgrounds. Due to the difficulties, traditional object detection methods often struggle to accurately identify small targets or targets within complex backgrounds. Therefore, we employed Dynamic Head [17] to promote the performance on the small targets detection.

**Figure 6:**
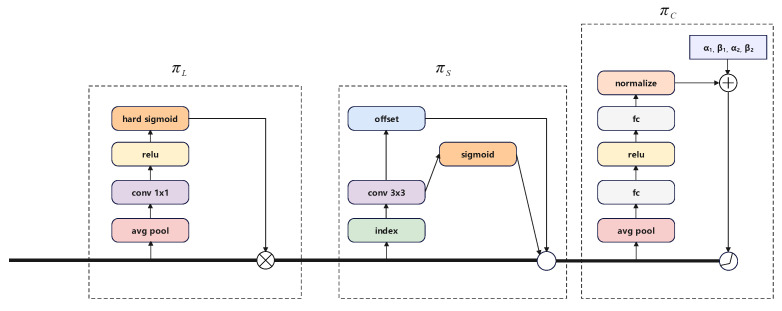
The Dynamic Head incorporates three attention mechanisms: Scale-aware, Spatial-aware, and Task-aware attentions. This diagram shows the process of dynamically adjusting feature emphasis on different scales, spatial regions, and task-specific features to enhance object detection performance.

In traditional Feature Pyramid Networks [34], $F\in R^{L\times H\times W\times C}$ represents the features across all levels of the feature pyramid, where *L* represents the number of layers, and H,W,C represent the height, width, and channels. In the Dynamic Head, we define S=W×H so the tensor in the Feature Pyramid Network is reshaped into $F\in R^{L\times S\times C}$. Scale-aware, Spatial-aware, and Task-aware attention mechanisms are incorporated in the Dynamic Head. Their structures are illustrated in Figure 6.

Scale-aware Attention dynamically integrates features from various scales by depending on the significance, adjusting them according to their semantic importance. This attention mechanism can effectively improve performance issues in object detection caused by differences in object scales. The specific calculation equation is as shown in Equation (4).

(4) $$\pi_{L}\left(F\right)\cdot F=\delta \left(f\left(\frac{1}{SC}\sum_{S,C}F\right)\right)\cdot F$$

Here, f(·) is a convolutional layer close to 1×1, and δ is an activation function, with the specific expression being $\delta =max(0,\frac{x+1}{2})$, *F* represents the input feature map.

Spatial-aware Attention, designed with reference to DCN [36,37], is an attention mechanism in spatiality, which enables sparse sampling of features at different levels. It can unify features of different positions and levels. The specific calculation equation is shown in Equation (5).

(5) $$\pi_{s}\left(F\right)\cdot F=\frac{1}{L}\sum_{l=1}^{L}\sum_{k=1}^{K}w_{l,k}\cdot F\left(l,p_{k}+\Delta p_{k},c\right)\cdot \Delta m_{k}$$

Here, *L* stands for the level of the FPN, *w* is a module similar to convolution for multidimensional feature sampling used for feature collection, and *F* represents the input feature. *c* represents the sampled channel. *K* represents the area for sparse feature sampling, while $\Delta p_{k}$ and $\Delta m_{k}$, respectively, represent the position’s offset and the position’s weight, both of which are learned from the input feature map.

Task-aware Attention is a dynamic, task-sensitive attention mechanism that dynamically turns off and on the features of certain channels in different tasks. The specific calculation equation is shown in Equation (6).

(6) $$\pi_{C}\left(F\right)\cdot F=max\left(\alpha_{1}\left(F\right)\cdot F_{c}+\beta_{1}\left(F\right),\alpha_{2}\left(F\right)\cdot F_{c}+\beta_{2}\left(F\right)\right)$$

Here, $F_{c}$ stands for the features in the $channel_{c}$, *F* is the input feature map. $\alpha_{1},\alpha_{2},\beta_{1},\beta_{2}$ are initially set to [1, 0, 0, 0], and their final values are learned during the training process from the preceding feature maps. To diminish dimensions, it first performs global average pooling on the channel, followed by the application of two fully connected layers and a normalization layer. Ultimately, it utilizes a shifted sigmoid function to standardize the output to the range of [−1, 1].

**Figure 7:**
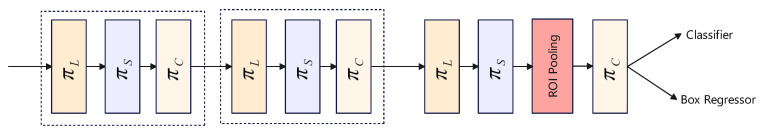
The structure of Dynamic Head, showing how it combines attention mechanisms to decide on object classes and their locations in the image.

The entire structure of Dynamic Head is shown in Figure 7. It consists of a collection of several attention structure modules, and an ROI Pooling layer [43]. At last, a decoupled detection head is used to output information related to categories and detection boxes separately.

OD-YOLO uses Dynamic Head as its detection head to greatly improve its ability to recognize targets from remote sensing images. This is particularly effective for small targets and complex backgrounds. Dynamic Head’s special attention mechanism offers better feature details, making the model more adaptable to changes in size and more focused on important areas. This boosts the detection quality. The enhancements not only improve accuracy on remote sensing images, but also make the model better at identifying targets in complex settings.

### 3.3. OIoU Loss Function

In the tasks of detecting objects, the difference between the model’s predictions and the true target is described by the loss function. It plays a crucial role as the model learns to be accurate. During the process of remote sensing target detection, the objects detected are typically small in size; therefore, accurately describing the detection boxes is quite challenging. When dealing with small objects, traditional loss functions might have some issues. Since small objects usually occupy fewer pixels in an image, traditional loss functions are quite sensitive to the pixel shift between predicted and true target boxes, which can lead to unstable results. Therefore, we propose OIoU loss function to precisely detect boxes. Its calculation process mainly includes the following parts:

#### 3.3.1. Angle Loss

**Figure 8:**
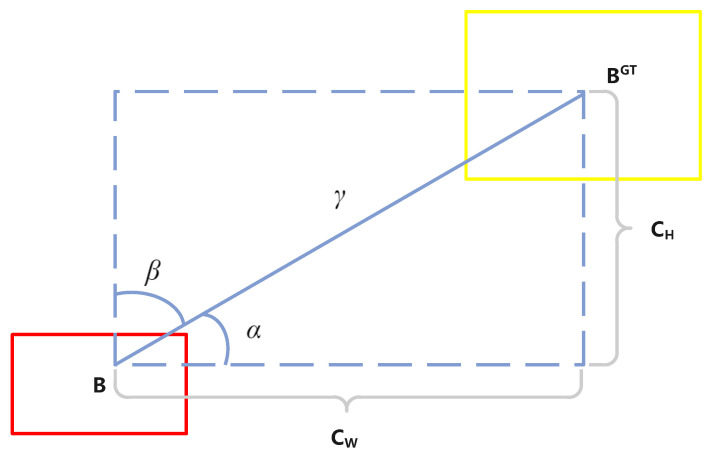
Diagram showing how angle loss is calculated between the predicted and real object boxes.

We use δ to represent the size of angle loss in OIoU. In this paper, *B* signifies the box predicted, and $B^{GT}$ signifies the real detection box, as shown in Figure 8. The definition of δ is shown in Equation (7).

(7) $$\delta =1-2\times {sin}^{2}\left(arcsin\left(\frac{C_{h}}{\gamma }\right)-\frac{\pi }{4}\right)=cos\left(2\times arcsin\left(\frac{C_{h}}{\gamma }\right)-\frac{\pi }{4}\right)$$

where $C_{h}$ is the height disparity between the predicted and true detection boxes, and γ represents the Euclidean distance between the centers of the two detection boxes. Throughout the training phase, if $\alpha \leq \frac{\pi }{4}$ minimize α first; if not, focus on reducing β.

Equation (7) describes the angular difference between the two detection boxes. If α is $\frac{\pi }{2}$ or 0 during training, the angle loss is 0. In the convergence process, if $\alpha \leq \frac{\pi }{4}$, minimizing α will be prioritized; otherwise, minimizing β will be prioritized.

#### 3.3.2. Distance and Shape Loss

**Figure 9:**
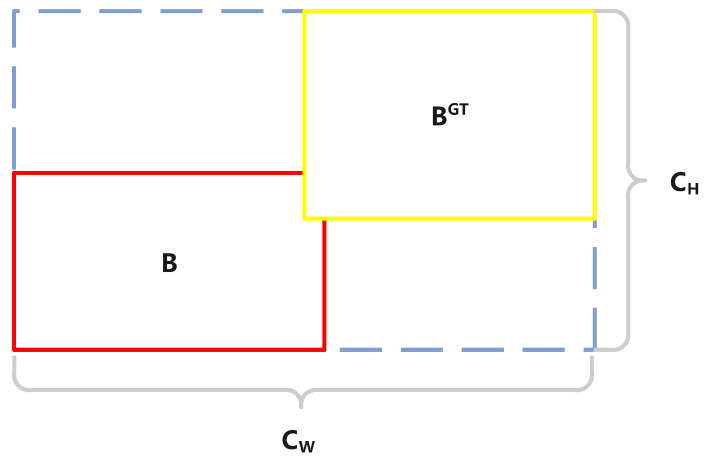
This shows how distance and shape loss is calculated between the predicted and real object boxes.

In OIoU, we define the distance loss as ϵ. As shown in Figure 9, $B^{GT}$ represents the real box, and *B* represents the predicted box. $B_{c}$ and $B_{c}^{GT}$. represent the centers of the two detection boxes, respectively. The calculation equation for ϵ is shown in Equations (8) and (9).

(8) $$\epsilon =\sum_{t=x,y}\left(1-e^{-\mu \rho_{t}}\right)=2-e^{-\mu \rho_{x}}-e^{-\mu \rho_{y}}$$

Here, the expressions of $\rho_{x}$ and $\rho_{y}$ are in Equation (9).

(9) $$\rho_{x}={\left(\frac{B_{c_{x}}^{GT}-B_{c_{x}}}{C_{w}}\right)}^{2},\rho_{y}={\left(\frac{B_{c_{y}}^{GT}-B_{c_{y}}}{C_{h}}\right)}^{2},\mu =2-\delta$$

Equations (8) and (9) show the discrepancy in the distance of the midpoints of the predicted and actual boxes. These equations take into account the Euclidean distance in both the x and y directions and use an exponential function to control the sensitivity of the loss. Here, μ is an adjustment parameter used to balance the impact of angle loss on distance loss. The goal of these equations is to bring the center of the predicted box as close as possible to the center of the real box, thus reducing the model’s error in target localization.

We also define the shape loss as ζ, with the specific calculation equation shown in Equations (10) and (11).

(10) $$\zeta =\sum_{t=w,h}{\left(1-e^{-w_{t}}\right)}^{\theta }={\left(1-e^{-W_{w}}\right)}^{\theta }+{\left(1-e^{-W_{h}}\right)}^{\theta }$$

Here, (w,h) and $\left(w^{GT},h^{GT}\right)$ stands for the width and height of the predicted and actual boxes. The parameter range of θ is [2,6], signifying the level of focus on shape loss, and it is set to 4 in the OD-YOLO. The expressions for $W_{w}$ and $W_{h}$ are shown in Equation (11).

(11) $$W_{w}=\frac{|w-w^{GT}|}{max(w,w^{GT})},W_{h}=\frac{|h\;-\;h^{GT}|}{max(h,h^{GT})}$$

Equations (10) and (11) measure the shape discrepancy between the predicted box and the real box. Through calculating the relative error in position of two boxes, and applying an exponential function to adjust the sensitivity of the loss, it adjusts the emphasis on shape loss. The goal of shape loss is to predict when true boxes are more closely aligned, improving detection accuracy.

In OIoU, there is another part of the distance loss that more accurately describes the variance in shape and distance in the predicted box and the real box. Here, we use η to represent it, with the specific calculation process shown as Equation (12).

(12) $$\eta =\frac{\sigma^{2}\left(b,b^{GT}\right)}{c^{2}}+\frac{\sigma^{2}\left(w,w^{GT}\right)}{C_{w}^{2}}+\frac{\sigma^{2}\left(h,h^{GT}\right)}{C_{h}^{2}}$$

Here, σ denotes the Euclidean distance of two points. $C_{w}$ and $C_{h}$ indicate the minimum bounding rectangle’s width and height for the predicted and real bounding boxes. *b* and $b^{GT}$ represent the centers of the two boxes, while (w,h) and $\left(w^{GT},h^{GT}\right)$ stand the width and height of the predicted and real boxes.

Equation (12) introduces a comprehensive distance loss, which scrutinizes the variations in shape and center position more closely between the predicted boxes and the actual ones. It calculates the square of the discrepancies in the shape of the boxes and center points between the predicted and real boxes. Then it is divided by the width and height of the minimum bounding rectangle that encloses both the predicted and actual boxes. It measures the differences in size and position between the two boxes. This design aims to more accurately measure and optimize the differences between the predicted and real boxes, further boosting the model’s effectiveness in detecting objects within remote sensing imagery.

#### 3.3.3. OIoU

**Figure 10:**
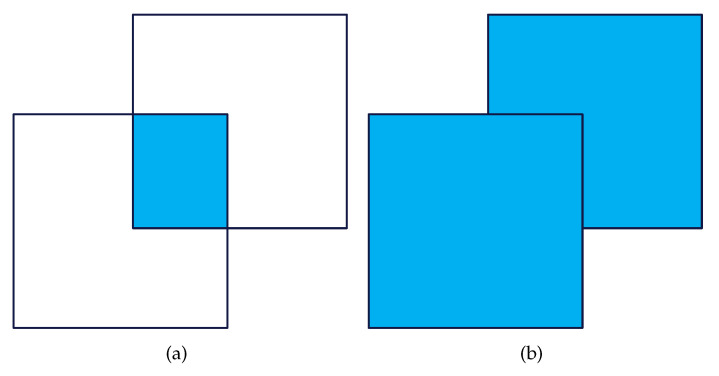
Illustration of the Intersection over Union (IoU) calculation for object detection. (**a**) Intersection; (**b**) Union.

In OIoU, we use IoU to describe the difference between predicted and real detection boxes. Figure 10a shows the intersection between the detection boxes, and Figure 10b shows their union. We use *A* to represent the predicted detection box, and *B* to represent the real detection box. The calculation equation for IoU is shown in Equation (13).

(13) $$IoU=\frac{A⋂B}{A⋃B}$$

In summary, the OIoU loss function is calculated as in Equation (14).

(14) $${Loss}_{OIoU}=1-IoU+\frac{\epsilon +\zeta +\eta }{3}$$

By introducing the OIoU loss function, the OD-YOLO model can more accurately detect small objects in remote sensing images and also improve its ability to recognize objects against complex backgrounds. Experimental results show that OD-YOLO outperforms several advanced models on the VisDrone dataset, with significant improvements in both mAP50 and mAP75 metrics. This demonstrates the OIoU loss function’s efficiency and superiority in tasks involving object detection in remote sensing imagery.

## 4. Experiment

### 4.1. Experiment Details

In this paper, the image resolution is set to 640×640. We choose the Adam optimizer to fine-tune the parameters, setting an initial learning rate of 0.01 and a weight decay of 0.0005. All computational tasks, including training and testing, are executed on an NVIDIA RTX 4060 Laptop GPU, utilizing Pytorch 2.0.1 and CUDA 11.8. To efficiently manage local memory resources, the BatchSize is established at 8. We use Mosaic data augmentation [44] in a training period and merge the four images together.

### 4.2. Dataset

**Visdrone** We chose the challenging VisDrone-2019 dataset [45], which has 8599 images to evaluate our model. These images were taken by drones at various positions and altitudes. In this dataset, there are lots of photos with small detection targets, dynamic blur, and obstructions. These challenges help evaluate the model’s effectiveness. The dataset is by default divided into 6471 training images and 548 validation images, totaling about 540k detection boxes. The rest being used as the test set, which is not used for training or evaluation.

**Foggy Cityscapes** We chose a challenging dataset for adverse weather conditions: Foggy Cityscapes [46]. This dataset is based on Cityscapes and each image has been fogged with coefficients of 0.01, 0.02, and 0.005, providing valuable reference for object detection in adverse weather. The dataset consists of a total of 8925 training images and 1500 validation images.

### 4.3. Evaluation Metrics

This paper mainly uses mAP50 and mAP75 as the key indicators, and their calculation process is as follows. First, we need to calculate the average precision (AP). In mAP50, we consider a detection correct if the IoU between the predicted box and the ground truth box is greater than 50%. In mAP75, we consider a detection correct if the IoU is greater than 75%. After calculating the AP for each category, we take the average of the AP values for all categories to obtain the final mAP. The specific computational equation is as shown in Equation (15).

(15) $$mAP=\frac{1}{m}\int_{1}^{m}AP$$

### 4.4. Experimental Results

We performed a comparison analysis of our proposed framework against alternative methods, with the results presented in Table 1. OD-YOLO achieves 36.16% on mAP50 and 21.6% on mAP75. When compared with YOLOv8n, YOLOv5s, Cascade-RCNN, RefineDet, and M2S, it shows an improvement of 5.2%, 9.9%, 4.2%, 7.3%, and 6.4% on mAP50, and 4.4%, 7.4%, 6.6%, 7.5%, and 7.4% on mAP75. These results show that OD-YOLO not only detects more objects accurately on the VisDrone dataset for remote sensing images, but it is also better at detecting objects of varying scales and complexities. Especially at high IoU thresholds, OD-YOLO still maintains high detection accuracy. This means it locates and recognizes targets very accurately. It shows OD-YOLO’s strengths and potential in detecting targets.

Table 2 details the comparison between the OD-YOLO model and the YOLOv8n model in detecting different types of targets on the VisDrone dataset. It lists the average precision (AP) for 10 categories, as well as the mAP at IoU thresholds of 0.5 and 0.75. Thorough comparing the AP, mAP50 and mAP75 of YOLOv8n and OD-YOLO in each category, it is clear that OD-YOLO performs better in almost all categories. Specifically, OD-YOLO not only shows a clear improvement in precision when dealing with small target recognition tasks but also demonstrates higher detection accuracy at higher IoU thresholds. This enhancement validates the efficacy and promise of the OD-YOLO model for applications in remote sensing image analysis.

**Figure 11:**
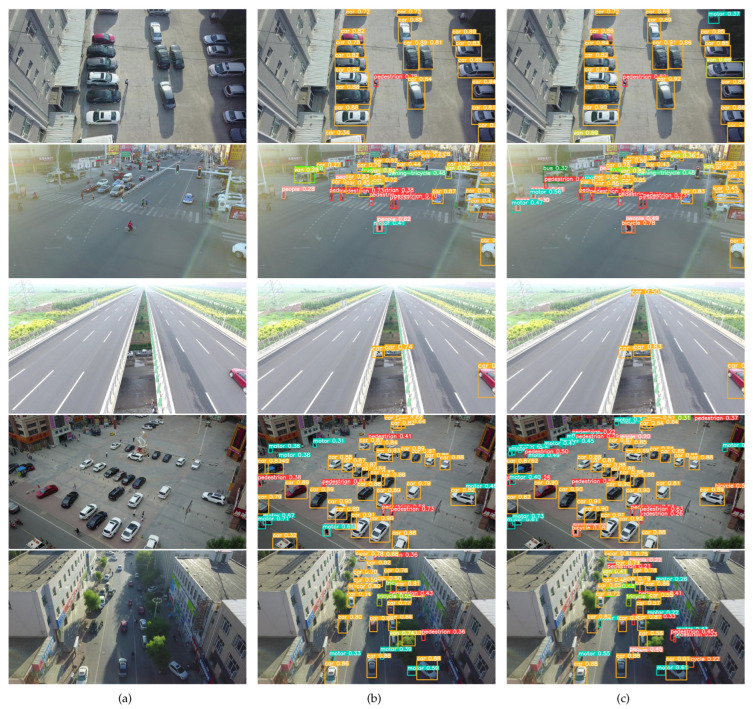
The detecting result comparison between YOLOv8n and OD-YOLO. (**a**) Original images. (**b**) The result of YOLOv8n. (**c**) The result of OD-YOLO.

Figure 11 showcases the detection outcomes for five representative images using YOLOv8n and OD-YOLO. From the illustrations, it is evident that OD-YOLO surpasses YOLOv8n in several key areas: it excels in identifying smaller objects, operates more effectively in dimly lit environments, and demonstrates superior performance in navigating complex roadways. OD-YOLO showcases heightened confidence in its target detections, with marked improvements in recognizing distant small targets and those obscured by shadows. Moreover, in intricate scenes, OD-YOLO achieves greater accuracy in classifying various categories and boasts a higher success rate in target identification. This enhanced capability is particularly pronounced in the model’s handling of small object recognition, where OD-YOLO consistently outperforms its predecessor. The advancements made by OD-YOLO can be attributed to its refined algorithms that better interpret the nuances of remote sensing imagery, including the intricate play of light and shadow, as well as the model’s adeptness at processing the detailed textures and shapes indicative of small and distant objects. This increased accuracy not only improves the reliability of the detections, but also minimizes the instances of false positives and negatives, crucial for applications requiring high precision.

**Figure 12:**
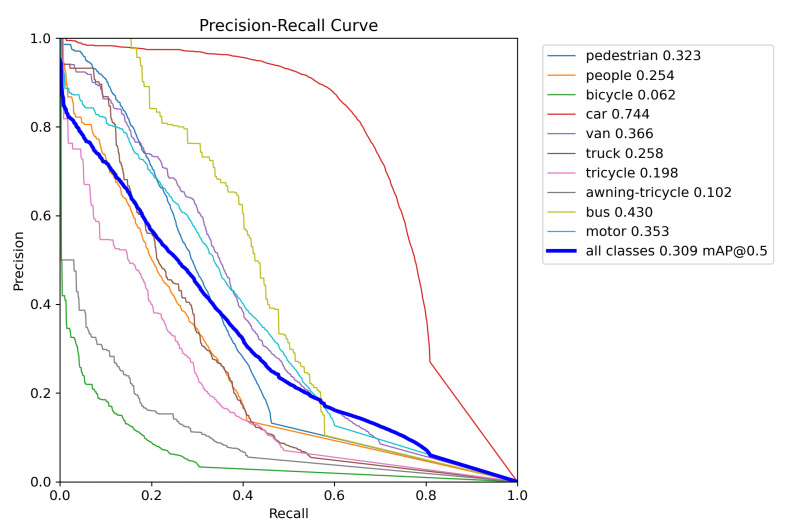
The Precision-Recall curve of YOLOv8n.

**Figure 13:**
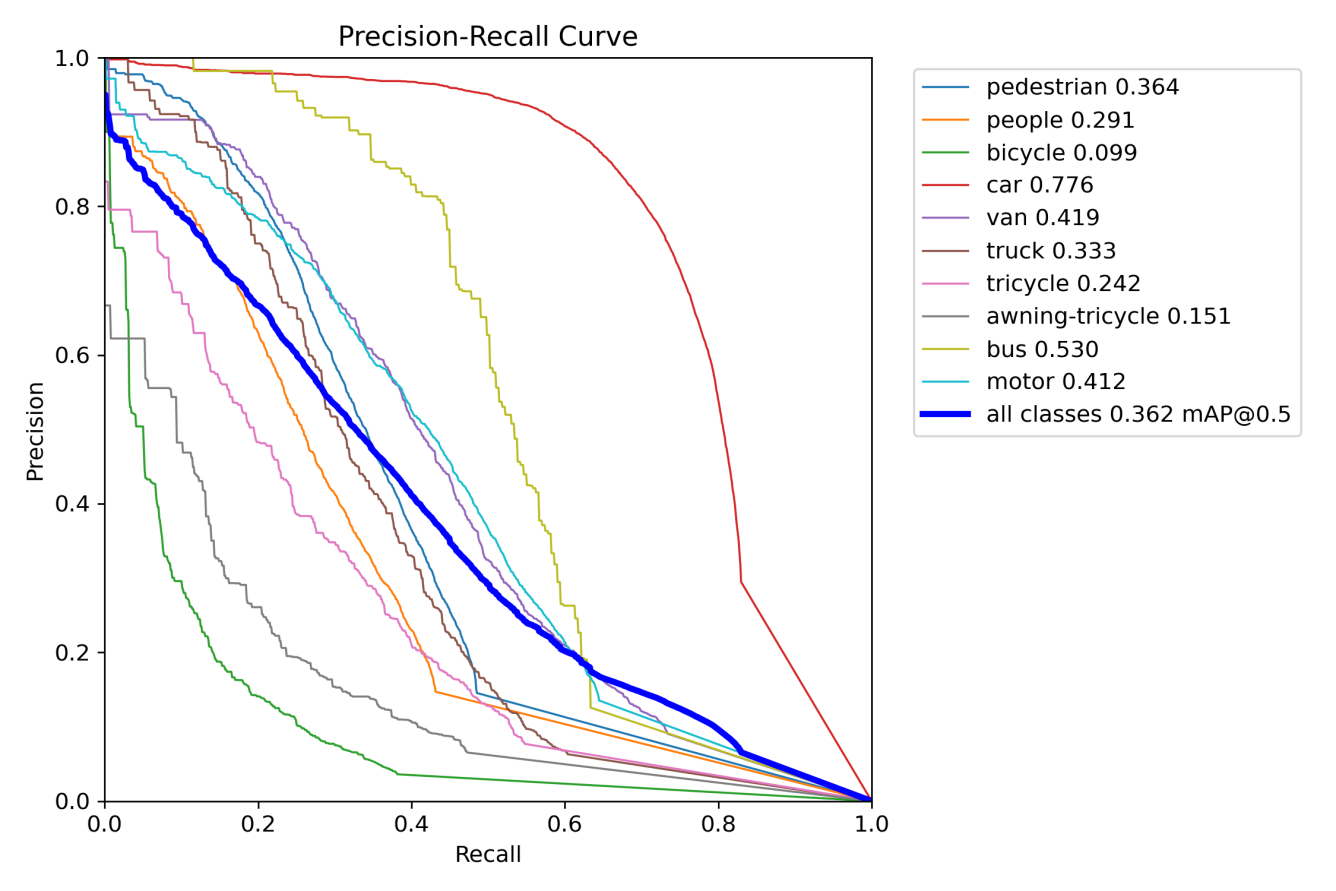
The Precision-Recall curve of OD-YOLO.

In order to evaluate the effectiveness of the model, this study plots the Precision–Recall curve. Each point on the curve represents the precision and recall at a specific threshold. The shape of the curve reflects the classifier’s performance at different thresholds. Generally, we aim for a high precision while maintaining a high recall, to ensure that all positive samples are identified as much as possible while minimizing false positives. Figure 12 shows the PR curve for YOLOv8n, and Figure 13 shows the PR curve for OD-YOLO. In the figures, it is evident that OD-YOLO achieves higher precision at the same recall level. This means the OD-YOLO model can provide more accurate predictions while maintaining a high recall rate. The Precision–Recall curve of OD-YOLO is smoother, indicating that the predictions from the OD-YOLO model are more stable and consistent.

**Figure 14:**
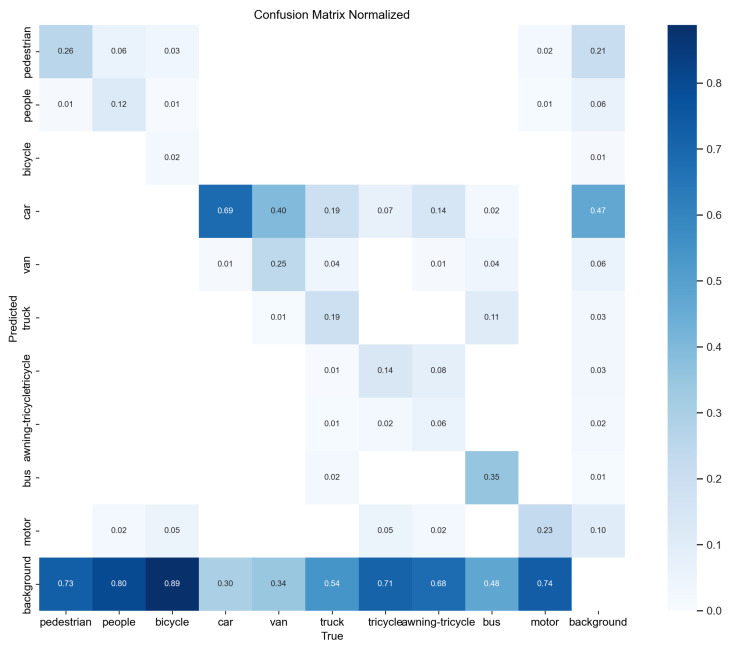
The confusion matrix of YOLOv8n.

**Figure 15:**
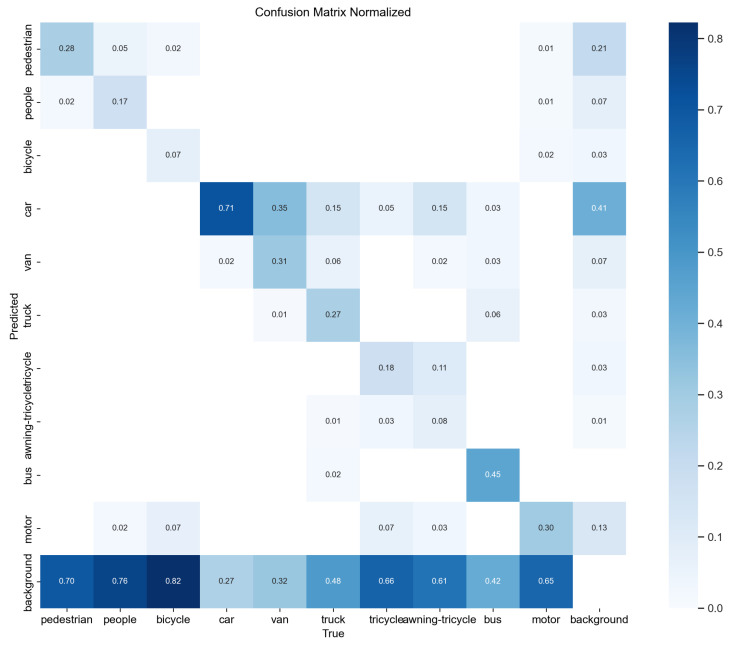
The confusion matrix of OD-YOLO.

A confusion matrix is a commonly used tool for evaluating classifiers in classification problems. It shows how well the classifier performs by organizing the prediction results into a matrix based on the true and predicted categories. This visually shows how the classifier does with different categories. Figure 14 represents the confusion matrix for YOLOv8n, and Figure 15 represents the confusion matrix for OD-YOLO. In the confusion matrix, the horizontal axis shows the true values, and the vertical axis shows the predicted values. By looking at the confusion matrices of both models, OD-YOLO has higher accuracy and lower error rates for each category, showing that it is better at correctly predicting multiple categories. Compared to YOLOv8n, OD-YOLO has fewer mix-ups and is more accurate in distinguishing samples of different categories. Overall, OD-YOLO’s confusion matrix is better than YOLOv8n’s, with more true positives, lower misclassification rates, higher precision, and fewer cases of mixing up categories.

**Figure 16:**
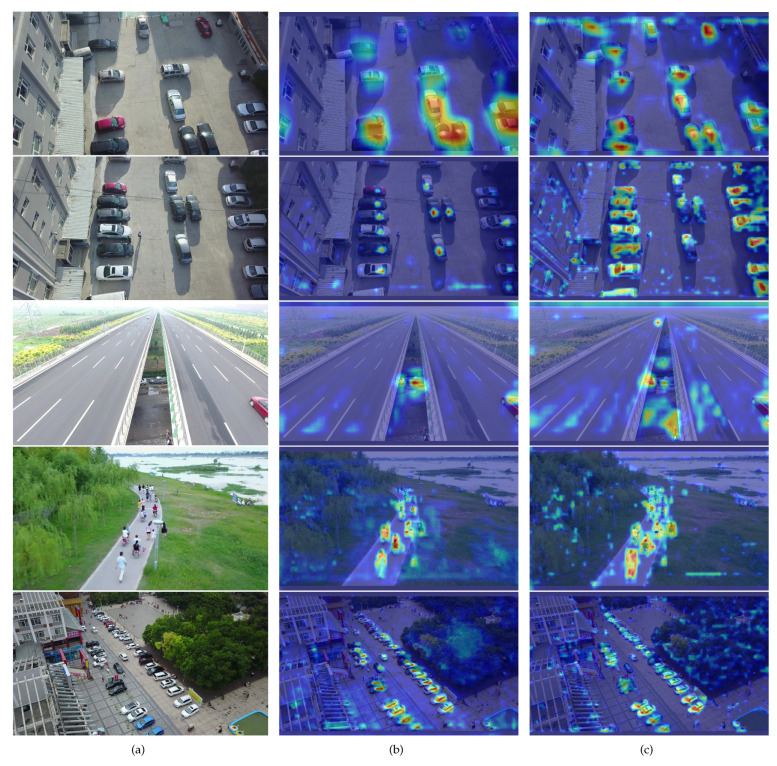
The figure displays a comparison of heatmaps between YOLOv8n and OD-YOLO. (**a**) is the original picture, (**b**) is the heatmap of YOLOv8n, and (**c**) is the heatmap of OD-YOLO. It can be observed in the figure that the colors representing small targets in OD-YOLO are deeper, indicating that OD-YOLO has a stronger capability for feature extraction.

In this paper, we use Grad-CAM [51] technology to visualize features. We chose the C2f layer before the 128×128×128 Dynamic Head of Figure 1. This layer integrates features from several smaller scales within the backbone, demonstrating the backbone’s ability to extract features for small objects. In the visualized heatmap, the deeper the color, the greater the contribution to the outcome. As shown in Figure 16, OD-YOLO demonstrates a significant improvement in the capability to derive features from small targets compared to YOLOv8n. These feature extraction capabilities come from the DRmodule’s superior ability to extract small-scale features, and OIoU helps the model better locate the position and shape of the objects.

To better verify the role of OIoU in small object detection, we used YOLOv8n as the training model and conducted experiments on the Visdrone dataset for comparison. In the standard YOLOv8n, the CIoU loss function is used. We conducted comparison experiments by replacing the loss function. As show in Table 3, the experiments showed that OIoU has a significant advantage in small object detection compared to other loss functions. It improved the AP metric by up to 1.4%, mAP50 by up to 1.8%, and mAP75 by up to 1.4%. This indicates that OIoU has a clear advantage in small object detection. This advantage comes from OIoU’s more accurate consideration of the angle and distance differences between the predicted box and the ground truth box, resulting in more accurate bounding box positioning.

To evaluate the performance of the OD-YOLO model in complex weather conditions, we chose the challenging Foggy Cityscapes dataset for comparison experiments. It is important to note that the Foggy Cityscapes dataset is derived from the Cityscapes dataset, and its labels also come from Cityscapes. However, because the Cityscapes dataset is suitable for both object detection and panoptic segmentation tasks, some class settings are not suitable for evaluating object detection. Therefore, we adopted the mainstream method for evaluating the model on Foggy Cityscapes: training with all classes together and selecting the average Precision (AP) of eight classes (person, rider, car, truck, bus, train, motorcycle, bicycle) and the mean AP of these eight classes for comparison.

Table 4 shows the performance of OD-YOLO and other models on the Foggy Cityscapes dataset. From the table, we can see that OD-YOLO has a significant advantage compared to other models. Compared to YOLOv8n, YOLOv5n, SIGMA, and DeFRCN, OD-YOLO shows a notable improvement in each category, with an overall mAP increase of 5.5%, 8%, 8%, and 20.4%, respectively, for the eight categories. Compared to MILA, although there is a slight decrease in the car, bus, and train categories, the overall mAP increased by 2.6%. The experimental results indicate that OD-YOLO significantly enhances feature extraction capability for object recognition tasks under adverse weather conditions.

**Figure 17:**
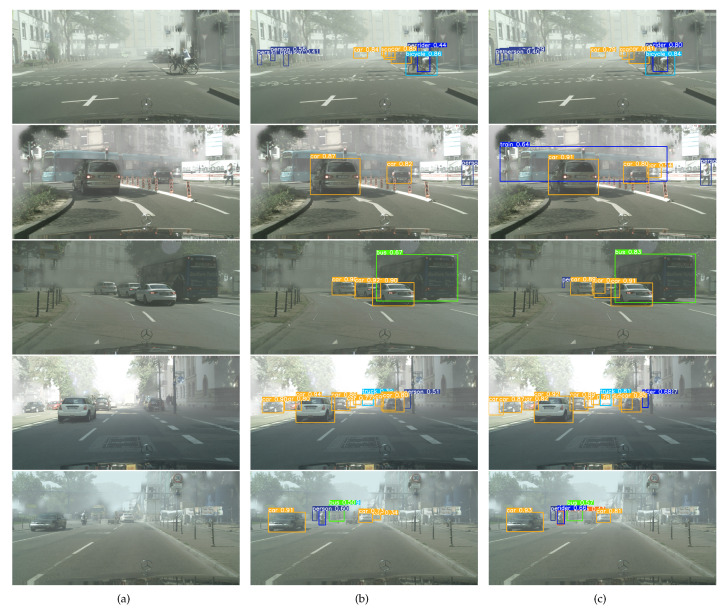
The detecting result comparison in Foggy Cityscapes dataset between YOLOv8n and OD-YOLO. (**a**) Original images; (**b**) The result of YOLOv8n; (**c**) The result of OD-YOLO.

Figure 17 shows a comparison of typical prediction results between YOLOv8n and OD-YOLO on the Foggy Cityscapes dataset. In the first image, YOLOv8n mistakenly detects a traffic sign as a person, while OD-YOLO better detects the person in dense fog conditions. In the second image, OD-YOLO accurately detects a train and a small, occluded car. In the third image, OD-YOLO successfully detects a car that is partially occluded by a nearby car and also accurately detects a person in dense fog conditions. In the fourth image, OD-YOLO accurately detects a nearby car covered in dense fog. In the fifth image, OD-YOLO avoids YOLOv8n’s mistake of identifying a traffic sign as a car and successfully detects a motorcycle. This demonstrates that OD-YOLO has better object detection performance under adverse weather conditions compared to YOLOv8n.The improvement in OD-YOLO’s detection capability under adverse weather conditions comes from the enhanced modules within OD-YOLO. These modules better extract the less obvious features of objects in adverse weather, particularly the DRmodule’s ability to extract blurred features. Such enhancements allow the model to meet higher accuracy requirements for object recognition in adverse weather conditions.

### 4.5. The Result of Ablation Study

To evaluate the effectiveness of each module and investigate their impact on the accuracy of the algorithm, we conducted a variety of ablation experiments. Starting with YOLOv8n as the baseline, we incrementally added improvements. According to the ablation experiment data in Table 5, incorporating the DRmodule into the backbone network improved the mAP50 and mAP75 by 1.3% and 0.9%, respectively, compared to the original network. This demonstrates that the DRmodule significantly enhances the model’s ability to capability to derive geometric shape features, boosting its performance. Adding the Dynamic Head module on top of the DRmodule further increased mAP50 and mAP75 by 3.1% and 2.6% indicated that Dynamic Head greatly improves the feature fusion ability in the feature pyramid of the YOLO model, achieving excellent results in object detection. Finally, including the OIoU loss function in the model resulted in an additional increase of 0.8% in mAP50 and 0.5% in mAP75, showing that OIoU accurately describes the prediction accuracy of small object detection, thereby enhancing model performance.

GFLOPs represent the number of floating-point operations required by the model, indicating its computational complexity. FPS represents the number of frames that can be detected per second. From the ablation experiments, OD-YOLO increased the computational complexity by 3.7 GFLOPs compared to YOLOv8n. Although the FPS decreased from 256.4 to 134.3, maintaining 134.3 FPS on devices with limited computing power still allows for very smooth object detection tasks. This drop in frame rate is completely justified.

## 5. Discussion

While the model demonstrates superior performance in small object detection within remote sensing images, there are several limitations and avenues for future work that merit attention. One of the primary limitations of the OD-YOLO model lies in its computational efficiency and resource requirements. The integration of complex modules like the DRmodule and the feature fusion detection head, while beneficial for accuracy, significantly increases the computational load. This can pose challenges for real-time applications or when deploying on hardware with limited processing capabilities. Secondly, object image recognition in remote sensing can be affected by objective factors such as weather. Although in this experiment, OD-YOLO performed very well under adverse weather conditions, there is still a need to explore and address object detection in extreme conditions.

Looking ahead, there are several promising directions for enhancing the OD-YOLO model. First, optimizing the model architecture to reduce computational demands while maintaining or even improving accuracy would make OD-YOLO more practical for a broader array of applications, including those requiring real-time processing. Investigating lightweight versions of the model that do not compromise significantly on performance could be particularly beneficial. For example, using lightweight methods such as GhostModule [59] to replace certain structures in the model can improve the model’s real-time performance while maintaining accuracy. Secondly, we will explore more data processing techniques. For example, in extreme weather conditions, we can use advanced image processing techniques such as dehazing [60]. In the future, we will focus more on researching image processing methods for remote sensing image object recognition, facilitating object detection under extreme conditions. Thirdly, to achieve better detection results, we will fine-tune our model to achieve optimal performance.

In conclusion, while the OD-YOLO model represents a significant step forward in remote sensing image analysis, continuous efforts in addressing its limitations and exploring future directions are essential for advancing the field and meeting the evolving demands of practical applications.

## 6. Conclusions

In this paper, we introduce the OD-YOLO model, designed specifically for the task of recognizing targets in remote sensing images. This model incorporates three key components: the DRmodule, which boosts feature fusion; a detection head that improves the feature pyramid structure; and the OIoU loss function, tailored to enhance recognition of small targets. Our experiments conducted on the VisDrone and Foggy Cityscapes dataset demonstrate that OD-YOLO outperforms existing models in detecting small targets and bad weather condition. Moving forward, we aim to further refine the model’s performance, striving to develop more effective target recognition approaches for remote sensing imagery.

## Figures and Tables

**Table 1:** The Comparsion of various state-of-the-art models on VisDrone dataset.

Model	AP	mAP50	mAP75
YOLOv8n	17.6%	30.9%	17.2%
YOLOv5n [47]	14.2%	25.1%	13.4%
YOLOv5s [47]	15.00%	26.6%	14.2%
Cascade-RCNN [48]	16.1%	31.9%	15.0%
RefineDet [49]	14.9%	28.8%	14.1%
M2S [50]	16.1%	29.7%	14.2%
**Ours**	21.8%	36.1%	21.6%

**Table 2:** The result of different classes on VisDrone dataset.

Class	YOLOv8n			OD-YOLO		
	AP	mAP50	mAP75	AP	mAP50	mAP75
pedestrian	0.135	0.323	0.0899	0.163	0.364	0.121
people	0.086	0.254	0.036	0.109	0.291	0.0538
bicycle	0.024	0.0615	0.015	0.041	0.0995	0.0266
car	0.508	0.744	0.563	0.546	0.776	0.607
van	0.251	0.366	0.286	0.297	0.419	0.339
truck	0.163	0.258	0.17	0.22	0.333	0.243
tricycle	0.107	0.198	0.106	0.134	0.242	0.212
awning-tricycle	0.0625	0.102	0.0653	0.0958	0.151	0.106
bus	0.282	0.43	0.309	0.374	0.53	0.431
motor	0.142	0.353	0.0841	0.176	0.412	0.115

**Table 3:** The Comparsion of various loss function on VisDrone dataset.

Loss Function	AP	mAP50	mAP75
CIoU [52]	17.6%	30.9%	17.2%
DIoU [53]	17.7%	31.1%	17.6%
EIoU [54]	17.2%	30.2%	17.0%
GIoU [55]	17.0%	30.0%	16.8%
WIoU [32]	17.3%	30.5%	17.2%
**OIoU**	18.4%	31.8%	18.2%

**Table 4:** The Comparsion of various state-of-the-art models on Foggy Cityscapes dataset.

Model	Person	Rider	Car	Truck	Bus	Train	Motorcycle	Bicycle	mAP
YOLOv8n	45.2%	65.4%	60.2%	35.1%	53.4%	26.6%	30.4%	55.2%	47.7%
YOLOv5n	42.9%	61%	58.6%	28.0%	52.8%	19.5%	39.9%	50.9%	44.2%
SIGMA [56]	44%	43.9%	60.3%	31.6%	50.4%	51.5%	31.7%	40.6%	44.2%
DeFRCN [57]	34.3%	41.4%	47.3%	24.3%	32.9%	17.3%	26.6%	38.4%	32.8%
MILA [58]	45.6%	52.8%	64.8%	34.7%	61.4%	54.1%	39.7%	51.5%	50.6%
**Ours**	47.5%	67.3%	63.2%	42.8%	56.5%	49.6%	41.9%	56.5%	53.2%

**Table 5:** The result of ablation study on VisDrone dataset.

Model	AP	mAP50	mAP75	GFLOPs	FPS
YOLOv8n	17.6%	30.9%	17.2%	8.9	256.4
+DRmodule	18.2%	32.2%	18.1%	10.4	214.3
+Dynamic Head	21.2%	35.3%	21.1%	12.6	134.3
+OIoU	21.8%	36.1%	21.6%	12.6	134.3

## Data Availability

We used the publicly available VisDrone2019 dataset. The url is https://github.com/VisDrone/VisDrone-Dataset (accessed on 5 September 2019). The code has been uploaded to https://github.com/LenterB/OD-YOLO (accessed on 29 May 2024).
